# Medication-related osteonecrosis of the jaw in an adult with osteogenesis imperfecta: a case report

**DOI:** 10.3389/froh.2026.1790684

**Published:** 2026-03-09

**Authors:** Wang-yong Zhu, Wenjun Wan, Ping-An Wu, Li-wei Zhou

**Affiliations:** 1 Department of Dental Surgery, The University of Hong Kong-Shenzhen Hospital, Shenzhen, Guangdong, China; 2 School of Stomatology, Jinan University, Guangzhou, China; 3 Department of Surgery, Division of Otolaryngology, Head and Neck Surgery, The University of Hong Kong-Shenzhen Hospital, Shenzhen, China; 4 Shenzhen Clinical Research Center for Rare Diseases, Shenzhen, China

**Keywords:** antiresorptive therapy, bisphosphonate, epistaxis, medication-related osteonecrosis of the jaw, oronasal fistula, osteogenesis imperfecta, pamidronate

## Abstract

Bisphosphonates (BPs) are widely used for managing tumor metastasis and bone diseases, associated with a rare complication of medication-related osteonecrosis of the jaws (MRONJ). Though reasons are still debated, the MRONJ occurrence in paediatric population suffering osteogenesis imperfecta (OI) and treated with BPs is absent. Here, we reported a 33-year-old patient with OI (Type III) who developed MRONJ after receiving intravenous pamidronate from 16 years old. She complained of intermittent nasal bleeding for six years, and presented an exposed necrotic bone in the left maxilla. She underwent surgical debridement, and pathological examination confirmed MRONJ. To our knowledge, this was a very rare case of MRONJ in an adult with OI type III following pediatric BP exposure. This case underscored the need for long-term vigilance, as delayed onset into adulthood is possible. Clinicians should pay attention to sinonasal symptoms as potential manifestations of MRONJ, and strengthen dental preventive care to reduce local risk factors. Long-term follow-up longitudinal studies with a larger sample size in this population are needed.

## Introduction

Medication-related osteonecrosis of the jaw (MRONJ) is a severe adverse effect of antiresorptive medications, such as bisphosphonates (BPs) and denosumab, predominantly reported in adults with osteoporosis or malignancy. Risk factors for MRONJ include dosage, route of administration, and duration of antiresorptive medication, invasive dental procedures, concurrent use of corticosteroids or immunosuppressants, poor oral hygiene, obesity, smoking, alcohol intake and concomitant diseases or conditions such as anemia and diabetes mellitus (1, 2). In adult populations, the incidence of MRONJ varies significantly depending on the underlying condition and type of medication. The frequency of MRONJ in osteoporosis patients receiving antiresorptive medications is estimated to be 0.001%–0.01%, and the frequency in oncology patients receiving higher doses is 1%–15% (3).

The occurrence of MRONJ in paediatric populations is considered exceptional, especially in patients with osteogenesis imperfecta (OI), an inherited collagen disorder caused by autosomal dominant mutations in genes encoding collagen type I (COL1A1 and COL1A2) and characterized by brittle bones, low bone mass, and increased fracture susceptibility (4). Although BPs are widely used to improve bone mass in OI, current literature reports few case of MRONJ and suggests a low risk of MRONJ in children and adolescents, attributed to high bone turnover and lower cumulative drug exposure (5). Here we present a case of MRONJ in an adult with OI following pamidronate therapy initiated during adolescence.

## Case report

**Figure 1:**
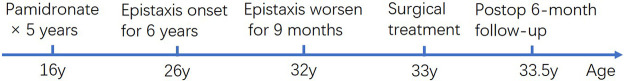
A timeline for the case presentation.

A 33-year-old female with OI (Type III) presented to the department of dental surgery, University of Hong Kong-Shenzhen Hospital. She complained of intermittent and spontaneous nasal bleeding for six years, worsening over the past nine months. She reported concurrent nasal obstruction, discomfort in anterior upper teeth, and intermittent purulent rhinorrhoea. The whole-genome molecular genetic testing report of the patient indicated that the likely pathogenic variant was a structural variant in the COL1A2 gene at the chromosomal locus chr7:94427443_94441723. For managing OI, she received annual intravenous pamidronate (unknown dosage) at age 16, with five doses. No subsequent use of other antiresorptives or antiangiogenics. She underwent bilateral tibial and femoral corrective osteotomies at age 23. No history of hypertension, diabetes mellitus, hepatitis, malignancy or radiation therapy. No history of dental extraction or denture restoration, no smoking or alcohol use. Her younger sister also has been diagnosed as OI. A timeline for the case was present in Figure 1.

Examination revealed mid-facial retrusion and Angle Class III malocclusion, with anterior open bite and crossbite, multiple missing teeth (#17, 16, 15, 13, 21–24, 26, 27, 33, 43), an inclined #25 with Grade 2 mobility, and a 1.5 cm × 1.0 cm exposed bone in the left maxillary premolar region, closing to #25. Oral hygiene was good.

**Figure 2:**
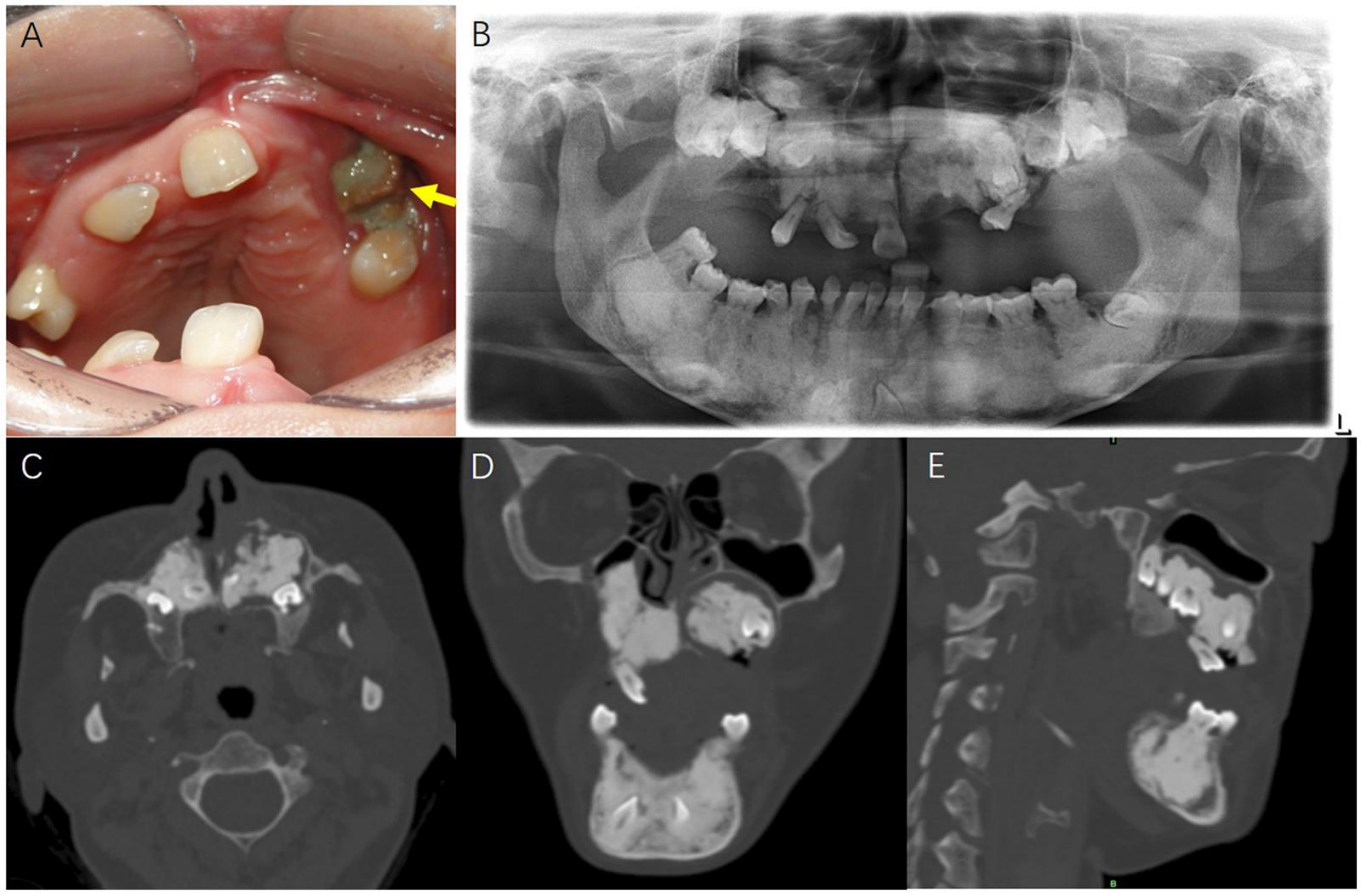
The representative intraoral photo and radiological images. **(A)** The intraoral image showed a 1.5 cm × 1.0 cm exposed bone (yellow arrow) in the left maxillary premolar region. **(B)** The pantomography showed diffuse, ill-defined radiopaque masses surrounding multiple impacted teeth in both maxilla and mandible. **(C–E)** The horizontal, coronal and sagittal CT images showed an extensive radiopaque mass in the left maxilla, surrounded by a radiolucent rim, with cortical dehiscence involved the nasal floor and buccal cortex.

Pantomography showed diffuse, ill-defined radiopaque masses surrounding multiple impacted teeth in both maxilla and mandible, and an alveolar bone absorption near the mesial root of #25. Computed tomography (CT) showed an extensive radiopaque mass in the left maxilla, surrounded by a radiolucent rim; cortical dehiscence involved the nasal floor and buccal cortex. Osteonecrosis of the jaw, cemento-osseous dysplasia–like changes, or odontogenic tumors was suspected. The representative intraoral photo and radiological images were presented in Figure 2. Laboratory tests were unremarkable.

Based on the history of BP exposure, clinical presentation of exposed bone in both nasal floor and oral cavity, a diagnosis of MRONJ [Stage 3: Exposed necrotic bone extending beyond the region of alveolar bone (1)] was made.

**Figure 3:**
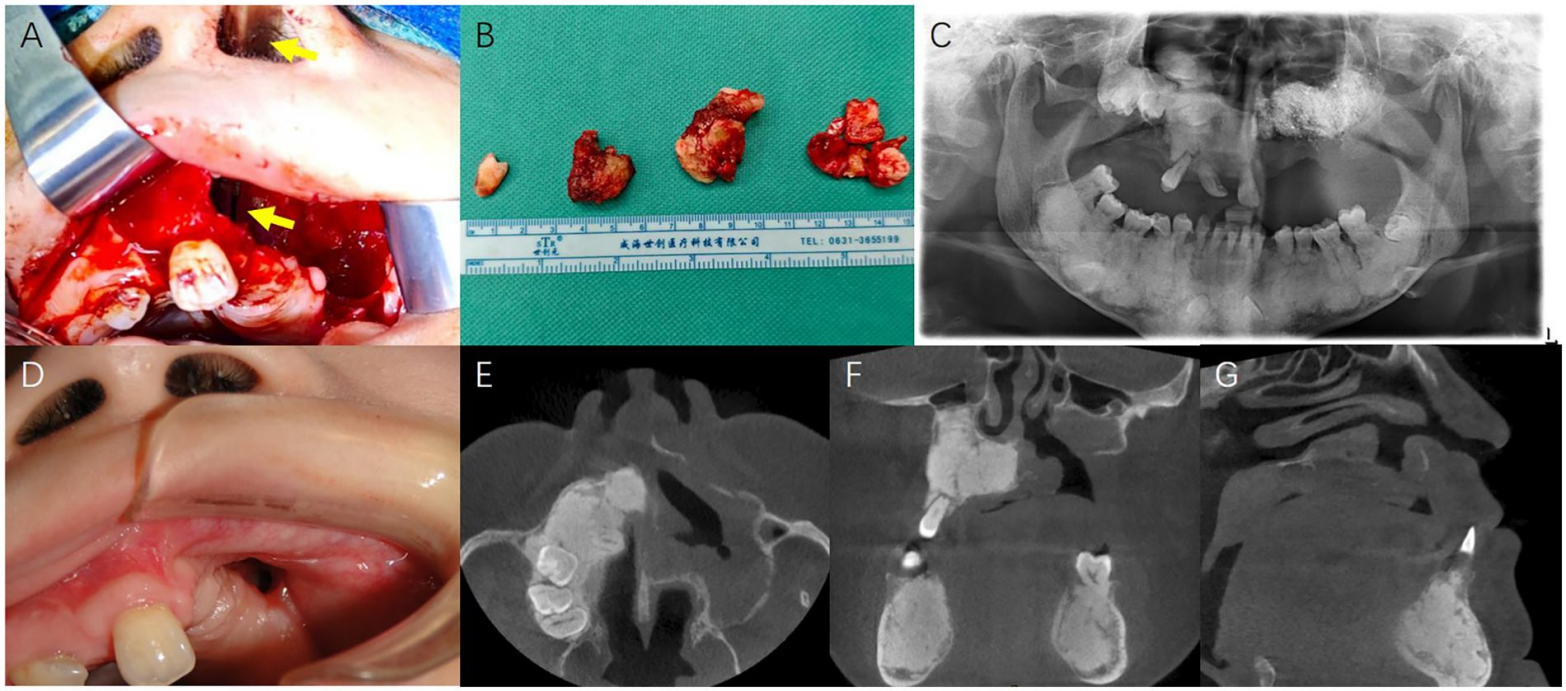
Intraoperative and postoperative photos. **(A)** The cavity after sequestrectomy of left maxillary necrotic bone, connecting with left nasal floor (yellow arrow pointing a vascular forceps). The fistula in left nasal floor explained the symptoms of intermittent and spontaneous nasal bleeding and concurrent nasal obstruction. **(B)** The yellow-grey necrotic bones containing impacted teeth was resected as specimen. **(C)** The postoperative pantomography showed the left maxillary cavity after sequestrectomy, packed with iodoform gauze. **(D)** During the 6-month postoperative follow-up, the intraoral image showed an oronasal fistula in the left maxilla. **(E–G)** The horizontal, coronal and sagittal CT images showed an oronasal fistula after sequestrectomy.

Under general anaesthesia, a full-thickness mucoperiosteal flap was elevated in the left maxilla. Avascular, yellow-grey necrotic bone containing impacted teeth was resected. Intraoperative frozen section excluded malignancy. A fistula in left nasal floor connecting to the maxillary cavity after sequestrectomy was confirmed by a nasal endoscope. The cavity was copiously irrigated with sterile saline and hydrogen peroxide, packed with iodoform gauze. The wound was closed primarily with watertight sutures. Perioperative antibiotic regimen included intravenous cefuroxime 1.5 g preoperatively, continued for 7 days postoperatively. The patient was instructed to use 0.12% chlorhexidine gluconate mouthwash twice daily commencing 24 h post-surgery. Tissue culture of the necrotic bone revealed *Neisseria subflava/Neisseria flavescens* group, *Neisseria sicca* group, *Eikenella corrodens*, *Streptococcus mitis* group, and *Haemophilus parainfluenzae*. Pathological examination of all specimens confirmed the diagnosis of MRONJ. The specimen comprised acellular necrotic lamellar bone devoid of osteocytes and osteoblasts. Adjacent soft tissue exhibited ulcerated stratified squamous epithelium with oedema, and a dense polymorphic infiltrate rich in plasma cells and lymphocytes. Postoperative recovery proceeded without acute complications; however, the patient was left with an oronasal fistula. During 6 months postoperative follow-up, there was no obvious infection or purulent discharge from the left maxillary oronasal fistula, and the patient had no subjective functional impairments such as unclear speech or food reflux. The patient has been referred for prosthetic consultation regarding an obturator. Longer-term monitoring was ongoing for every 3 months. Intraoperative and postoperative photos were presented in Figure 3.

## Discussion

This study reported a rare case of MRONJ in an adult patient with OI (Type III) following antiresorptive therapy. Although previous studies have suggested a low risk of MRONJ in pediatric and adolescent OI patients, this case highlighted that this rare complication remains a potential concern in the population of OI.

Several systematic reviews and retrospective studies have consistently reported the absence of MRONJ in pediatric and adolescent patients with OI treated with antiresorptive medications, suggesting a favorable safety profile in this population (5–7). Proposed explanations for this absence include the high bone remodeling capacity in children, lower cumulative drug dosages and shorter duration compared to adult pathologies (cancer metastases, osteoporosis, and other bone diseases), and the absence of age-related comorbidities such as diabetes or periodontal disease. Additionally, the unique skeletal characteristics of pediatric patients, such as thinner cortical bone with less bone mass in cancellous bone, higher vascularity, and more active bone remodeling, may contribute to a protective effect against MRONJ. However, these conclusions are limited by relatively short follow-up periods (the longest follow-up period was 5 years 8) and the rarity of OI, which restricts sample sizes and long-term data availability. Moreover, while no MRONJ cases have been reported in pediatric OI patients, the possibility of delayed onset in adulthood, especially as comorbidities develop, remains an open question requiring a longer-term follow-up.

Despite the absence of MRONJ in OI patients, there were a few MRONJ cases reported in pediatric and adolescent subjects receiving antiresorptive therapy for other conditions. Bredell et al. reported a 18-year-old woman with giant cell granuloma (GCG) who received denosumab therapy for 8 months and developed stage-2 MRONJ following a debulking surgery and intralesional steroid injection (9). Uday et al. reported a 19-year-old man with GCG underwent 46 denosumab doses (for 4 years), who experienced stage-2 MRONJ after dental extraction and required sequestrectomy (10). Innes-Taylor et al. reported a 9-year-old girl with an aneurysmal bone cyst who had been on intravenous denosumab for 14 months, developed stage-2 MRONJ 9 months after dental extraction of #46 and #85 (11). Moeini et al. reviewed radiographic signs of 12 patients between 7 and 21 year old (average 13 years) with MRONJ, who received BP therapy, while indications for the BP therapy were not available (12). These data indicate that while MRONJ remains rare in childhood, it can occur under specific clinical circumstances, particularly when high-potency antiresorptive medications are combined with invasive dental procedures.

This case fulfilled the 2022 AAOMS diagnostic criteria for MRONJ: presence of exposed necrotic bone for more than 8 weeks, a history of antiresorptive therapy, and no history of radiation or metastatic disease involving the jaws (1). However, the exact triggering cause for her MRONJ remained unclear. Since #25 is the only tooth on the left maxilla bearing all the biting forces, which showed a severe inclination and mesial alveolar bone absorption in pantomography, we proposed a hypothesis that occlusal trauma-induced periodontal inflammation in #25 might be the onset trigger of MRONJ, and then the inflammatory fistula in left nasal floor led to the intermittent and spontaneous nasal bleeding. Notably, this patient exhibited diffuse sclerosis of the maxilla and mandible, and multiple impacted teeth in radiographic examinations. Since most of these teeth should have erupted by 12 years of age, and her antiresorptive therapy was initiated at 16 years of age, we suppose that this may be a dental developmental anomaly occurring during the growth and development of pediatric patients with OI. For Stage 3 MRONJ, surgical debridement (resection of necrotic bone and impacted teeth) is the core treatment plan, aiming to control infection, eliminate necrotic tissue, and reduce the risk of fistula formation (13, 14). Jaw reconstruction approaches for MRONJ, such as vascularized free fibula flap or iliac crest flaps, is particularly challenging in OI patients due to abnormal bone architecture, compromised quality of blood vessels, and possible history of bone fractures and surgical reductions. In this case, secondary reconstruction following sequestrectomy was planned. However, her both fibulae were blade-like and had a history of fracture. The deep circumflex iliac artery flap might be a more suitable choice for her, which necessitated a highly individualized planning and approach. Besides, the exact pamidronate dosage and infusion intervals could not be retrieved from the patient's childhood medical records, which was a limitation for evaluating the risk of MRONJ onset in patient with OI.

## Conclusions

We reported a rare case of MRONJ in an adult with OI (Type III), who received adolescent intravenous pamidronate therapy. Although MRONJ is rare in OI patients, this case underscored the need for long-term vigilance, as delayed onset into adulthood is possible. Clinicians should pay attention to sinonasal symptoms (e.g., epistaxis, nasal obstruction) as potential manifestations of maxillary MRONJ, and strengthen dental preventive care to reduce local traumatic/inflammatory triggers. Long-term follow-up longitudinal studies with a larger sample size in this population are needed, to evaluate the association of cumulative BP doses in adolescence and MRONJ risk in adulthood.

## Data Availability

The raw data supporting the conclusions of this article will be made available by the authors, without undue reservation.
